# Possible Differential Diagnosis of the Degrees of Rheological Disturbances in Patients with Type 2 Diabetes Mellitus by Dielectrophoresis of Erythrocytes

**DOI:** 10.3390/jpm10030060

**Published:** 2020-07-05

**Authors:** Margarita V. Kruchinina, Andrey A. Gromov, Vladimir M. Generalov, Vladimir N. Kruchinin

**Affiliations:** 1 Research Institute of Internal and Preventive Medicine–Branch of the Institute of Cytology and Genetics, Siberian Branch of the Russian Academy of Sciences, B. Bogatkova Str., 175/1, 630089 Novosibirsk, Russia; gromov.center@rambler.ru; 2 Federal Budgetary Research Institution “State Research Center of Virology and Biotechnology Vector”, Rospotrebnadzor, 630559 Koltsovo Novosibirsk Region, Russia; general@vector.nsc.ru; 3 Rzhanov Institute of Semiconductor Physics Siberian Branch of Russian Academy of Sciences, Lavrentiev Ave., 13, 630090 Novosibirsk, Russia; vladd.kruch@yandex.ru

**Keywords:** diagnostic method, severity of disturbances, rheology, diabetes mellitus, erythrocyte, dielectrophoresis

## Abstract

Hemorheological disorders in structural and functional parameters of erythrocytes are involved in the pathological process in type 2 diabetes mellitus (DM). Aim: to investigate the feasibility of differential diagnosis of the degrees of rheological disturbances in patients with type 2 DM by dielectrophoresis of erythrocytes. Methods: 62 subjects (58.7 ± 1.6 years) with type 2 DM diagnosed according to the criteria of the ADA were subdivided into two groups: medium (*n* = 47) and high (*n* = 15) risk of microcirculatory disturbances (EASD, 2013). Electric and viscoelastic parameters of erythrocytes were determined by dielectrophoresis using an electric optical system of cell detection. Results: the progression of rheological disturbances in the patients with type 2 DM was accompanied by significant decreases in deformation amplitude; dipole moment; polarizability; and membrane capacity; and increases in conductivity, viscosity, rigidity, hemolysis, and formation of aggregates (*p* < 0.05). Combined use of the parameters increased sensitivity (97.8%) and specificity (86.7%) for diagnosis of rheological disturbances in type 2 DM. Conclusion: the proposed experimental approach possesses low invasiveness, high productivity, shorter duration, vividness of the results. The method allows to evaluate not only local (renal and ocular) but also systemic status of microcirculation using more than 20 parameters of erythrocytes.

## 1. Introduction

Among patients with diabetes mellitus (DM), early disability and mortality, which are mostly caused by diabetic angiopathies, are the most important socioclinical problem of modern diabetology [1]. Timely detection of microcirculatory disturbances and reversal of hemorheological disorders are currently recognized as major components of modern diagnostic and therapeutic strategies in relation to patients with DM.

At present, in clinical practice, physicians use a variety of methods for the detection of microcirculatory disturbances in DM.

There are methods for the detection of diabetic microangiopathy that are designed strictly for local vascular analysis (e.g., in kidneys and eyes). To detect diabetic retinopathy, clinicians utilize several methods: slit-lamp examination of the conjunctiva, fundoscopy, retinal fluorescent angiography, quantitative perimetry, autocampigraphy, and examination of darkness adaptation. These methods allow visual evaluation of the state of retinal vessels and extrapolation of the obtained results to the vascular system as a whole. Nonetheless, these methods require specially trained personnel and ophthalmological equipment. The shortcomings of direct ophthalmoscopy are a small examined area, the absence of stereoscopy, close contact with a patient, and the inability to examine the extreme periphery of the fundus. In clinical practice, this approach is convenient as a screening method of medical examination. Contact binocular ophthalmoscopy by means of a slit lamp and contact lenses is the golden standard of diagnosis of fundus pathologies. Nevertheless, this method has limitations related to inflammatory processes on the eye surface, well-pronounced opacification or degenerative alterations of the cornea, and health state of the patient (e.g., convulsive disorder or epilepsy) [2]. A microalbuminuria test, urinary sediment analysis, measurement of glomerular filtration using endogenous creatinine, and other assays that only indirectly evaluate the presence of microvascular changes in kidneys during DM, are more informative at later stages, necessitate a set of expensive equipment, are costly, and require specially trained personnel [3].

Currently, the methods of capillaroscopy and capillarography and determination of vascular permeability allow us to answer the question about the systemic status of capillaries. Furthermore, capillaroscopy together with biochemical indicators is a cornerstone for diagnosing the preclinical stage of diabetic microangiopathy. In case of a combinatorial approach to the assessment of the microcirculatory bed, clinicians perform capillaroscopy in a resting state with subsequent evaluation of structural changes in capillary status. Additionally, they perform capillaroscopy and oxyhemometric analysis with four functional trials and the action of physical stimuli on the extremity being analyzed: cuff occlusion, assessment of cold exposure, assessment of heat exposure, and testing of raising the extremity. This technique ensures the most accurate diagnosis of microangiopathies in this group of patients because of comprehensive evaluation of the microcirculatory bed at early stages of a disorder on the basis of measuring the reserve properties of capillaries [4]. It should be noted, however, that these methods lengthen medical examination, and their results are affected by several factors (ingestion of liquids, food, or alcohol as well as cigarette smoking, vibration, or some medications) that alter the capillary state of extremities (fungal lesions of nail phalanx tissues, burns, traumatic or mechanical injury of hands (or finger cuticles) or feet or impaired light scattering by finger skin as a result of damage by aggressive chemicals) [5,6].

Abroad, in patients with DM, clinicians employ the method of microangiopathy diagnosis [7,8] that consists of examination of nail bed capillaries in ring fingers of patients at rest and assessment of their structural alterations by measuring the main capillaroscopic parameters using a computer-based capillaroscope equipped with a camera. This approach involves resting-state capillaroscopy with subsequent evaluation of structural alterations of the capillary state. A drawback of this technique is that the well-known method does not take full advantage of the diagnostic algorithm, thus limiting its application at initial stages of microangiopathy in patients with DM. At the same time, the physician is unable to detect pathological changes of capillaries in patients with first-time DM or with short duration of the disease; this situation precludes (i) assessing the status of the microcirculatory-system reserve and the degree of hypoxia of peripheral tissues, (ii) identifying a microangiopathy stage, and ultimately (iii) monitoring the capillary state in a patient with DM as a function of time.

Histochemical and electron-microscopic analyses of blood vessels in skin biopsies enable the detection of characteristic-for-DM alterations in vessel walls in the form of basal-membrane thickening, proliferation of the endothelium, and increased deposition of PAS-positive substances. A biopsy can be taken from any site of the skin by means of a special dermatome, from an ear lobe, or from the mucous membrane of the mouth or rectum. Nonetheless, because of the complexity and certain traumaticity of the method, it is used not so much for diagnosis but rather for studying the pathogenesis and prevalence of diabetic microangiopathies [9].

Various types of proteomics have been employed for pathoanatomical research on diabetic microangiopathy. Common proteomic techniques, including gel-based ones, possess limited sensitivity and reproducibility and are usually used to detect biomarkers of high or medium prevalence. Clinical samples from patients with diabetic microangiopathy, e.g., biopsy samples, are rather small. Therefore, the technologies of sample preparation, quantitative labeling, and mass spectrometry should be optimized for detecting a protein at a low concentration, for processing of multiple samples, and for accurate quantitation. Besides, signal transduction pathways are difficult to study because they contain many low-abundance proteins. At present, there are no biomarkers identifiable by proteomic methods that can be applied to the diagnosis of diabetic microangiopathy. It is noteworthy that the high cost of proteomic methods is another obstacle to their widespread use in clinical settings [10].

Shortcomings and limitations of the methods described above necessitate the development of new approaches to the detection of microcirculatory disturbances in DM.

The pathogenesis of microvascular complications in type 2 DM is linked with the pathological involvement of erythrocytes (red blood cells, RBCs), which not only perform the oxygen transport function in the blood–tissue system but also participate in the regulation of rheological properties of the blood and in interactions with endothelial cells [11].

At the capillary level, the state of RBCs is a determinant of microcirculation. Moreover, many changes in blood fluidity, for instance, deformability and aggregation of RBCs, are detectable much earlier than the obvious systemic symptoms [12].

Microrheological properties of RBCs, such as deformability, the aggregation ability, and production of vasoreactive factors, are determined by specific features of the molecular organization of the erythrocytic membrane and of the peri-membrane cytoplasmic matrix [13,14].

The dielectrophoresis method proposed by us for studying the electric and viscoelastic properties of RBCs is based on changes in cell parameters under the action of a nonuniform alternating electric field (NUAEF). The use of different frequencies of the electric field helps to assay the state of both RBC membranes (low frequencies: 10^5^ and 5 × 10^4^ Hz) and cytoplasm (high frequencies: 10^6^ and 5 × 10^5^ Hz). A brief dielectrophoretic assay (1–2 min.) helps to determine many parameters (>20) reflecting structural and functional status of RBCs (e.g., their surface charge, deformability, membrane stability, propensity for aggregation, and electric conductivity of membranes). The above characteristics of RBCs are tightly associated with a pathological process, as revealed by us previously in patients with a diffuse pathology of the liver [15] or a cerebrovascular pathology [16].

Such dielectrophoresis-measured parameters as cell deformation amplitude, summarized viscosity, and summarized rigidity reflect membrane alterations and an increase in internal rigidity as a consequence of a high concentration of glycated hemoglobin (HbA1c). On the other hand, the velocity of cell motion toward electrodes, dipole moment, and cellular capacity denote blood serum biochemical shifts associated with DM (e.g., disturbances of lipid and purine metabolism). Polarizability of cells and levels of hemolysis and aggregation are related to altered conditions of RBC maturation processes and indicate their lowered resistance to various factors; this drawback will later affect their interaction with the cells of a changed endothelium.

The RBC parameters in patients with type 2 DM were studied here for the first time by dielectrophoresis. This approach has several substantial advantages over the existing assays of microcirculation: low invasiveness (only 10 μL of blood is sufficient, collected by a method customary for the patients); the ability to evaluate the microcirculation state in the body as a whole rather than at a few sites; the use of high technologies; vivid results (the physician can visualize RBC behavior in a NUAEF); low cost without the need for expensive reagents and equipment; high reproducibility of the results; and possible the diagnostics as well as personalization of the therapeutic regimen for a patient.

Therefore, electric and viscoelastic parameters of RBCs, as measured by dielectrophoresis, help to obtain valuable information that is directly related to the development of rheological disturbances in DM.

The objective of this work was to test the feasibility of differential diagnosis of degrees of rheological disturbances in patients with type 2 DM by means of RBC dielectrophoresis.

## 2. Materials and Methods

### 2.1. Partisipants

In the clinical diagnostic department of the Institute of Internal and Preventive Medicine during 2016, we recruited patients with type 2 DM in accordance with the criteria of the American Diabetes Association (2016) [17]. Our inclusion criteria were as follows: patients aged 25–70 years with type 2 DM, who signed written informed consent to participate in the study. The following exclusion criteria were applied: age younger than 25 or older than 70 years; a decompensated pathology of the cardiovascular, pulmonary, and/or digestive system; or refusal to sign the written informed consent to take part in the study.

Sixty-two patients with type 2 DM at age 58.7 ± 1.6 years (mean ± SEM) were analyzed; 43 (69.4%) of them were females and 19 (30.6%) were males. The healthy control group consisted of 38 subjects at age 48.5 ± 2.2 years (24 (63.2%) females and 14 (36.8%) males) without DM, and without any other detectable pathology of internal organs, who visited our clinical diagnostic department for preventive purposes (Group 1). The subjects in the control group led a healthy lifestyle, did not smoke, and did not drink alcohol more often than once or twice a month, and the alcohol doses did not exceed 40 g for males and 20 g for females in terms of pure ethanol. In this group, the electric and viscoelastic parameters of RBCs were within the reference range appropriate for respective age.

Patients with type 2 DM were subdivided into two groups: medium risk (Group 2) and high risk of microcirculatory disturbances (Group 3) according to various indicators in keeping with recommendations of the European Association for the Study of Diabetes (EASD) (2013) [18] and recommendations cited in references [19,20]:

- Glycosylated hemoglobin (HbA1c): <7% for the medium risk and >7% for the high risk;
- blood pressure: <140/85 mm Hg for the medium risk and >140/85 mm Hg for the high risk;
- High-density lipoprotein (HDL) cholesterol: >40 mg/dL for the medium risk and <40 mg/dL for the high risk;
- Low-density lipoprotein (LDL) cholesterol: <100 mg/dL for the medium risk and >100 mg/dL for the high risk.

The Biomedical Ethics Committee of the federal publicly funded institution Institute of Internal and Preventive Medicine (session of 18 December 2015) approved the study protocol.

The patients underwent comprehensive clinical examination and instrumental analyses, including electrocardiography (Cardiovit AT-10/AT-2 (Schiller AG, Baar, Switzerland); ultrasonography of abdominal-cavity organs, kidneys, heart, and vessels (Vivid 7 Dimension (GE HealthCare, Norway); fundoscopy (ophthalmoscope PanOptic 11810-CE Welch Allyn, Skaneateles Falls, NY, USA); an assay of glycated hemoglobin (NycoCard READER II (Alere Technologies AS, Norway); and determination of the albumin/creatinine ratio in spot urine [21].

At admission of in-patients, fasting collection of venous blood from the cubital vein was conducted in the amount of 8 mL into two vacutainers (Becton Dickinson, Burlington, North Carolina USA): the first one (5 mL; SST II Advance) for biochemical assays, and the second vacutainer (5 mL; containing 0.109 M sodium citrate, 3.2%, in the 9:1 ratio) for determining the electric and viscoelastic parameters of RBCs by dielectrophoresis.

Biochemical indicators (total cholesterol, HDL cholesterol, LDL cholesterol, triglycerides, fasting blood glucose, uric acid, creatinine, urea, AST activity, total bilirubin, and albumin) were determined using KONELAB PRIME 30i (Scientific Oy, Thermo Fisher, Vantaa, Finland). RBC parameters (the number of RBCs, the hemoglobin level, color indicator, hematocrit, mean corpuscular volume (MCV), mean corpuscular hemoglobin (MCH), mean cell hemoglobin concentration (MCHC), red cell distribution width (RDW-CV) in %, and red cell distribution width (RDW-SD) in femtoliters) and leukocyte counts were determined on an automatic hematology analyzer, HT Micro CC-20 Plus (High Technology, Inc., USA). The RBC sedimentation rate was measured by Panchenkov’s micromethod.

Blood fibrinogen was quantified by the Clauss method with reagents from Renam (Moscow, Russia). Immunoenzyme assays based on standard ELISA kits were carried out to quantitate serum C-reactive protein (Biomerica kit) on an immunoenzyme assay analyzer, Multiscan EX (Finland) [18].

### 2.2. Dielectrophoresis

**Figure A1:**
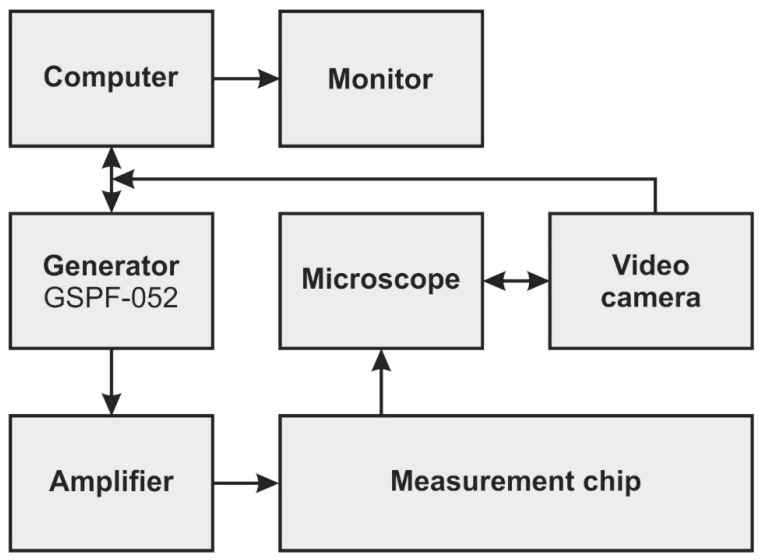
The functional scheme of the electric optical system of cell detection.

**Figure A2:**
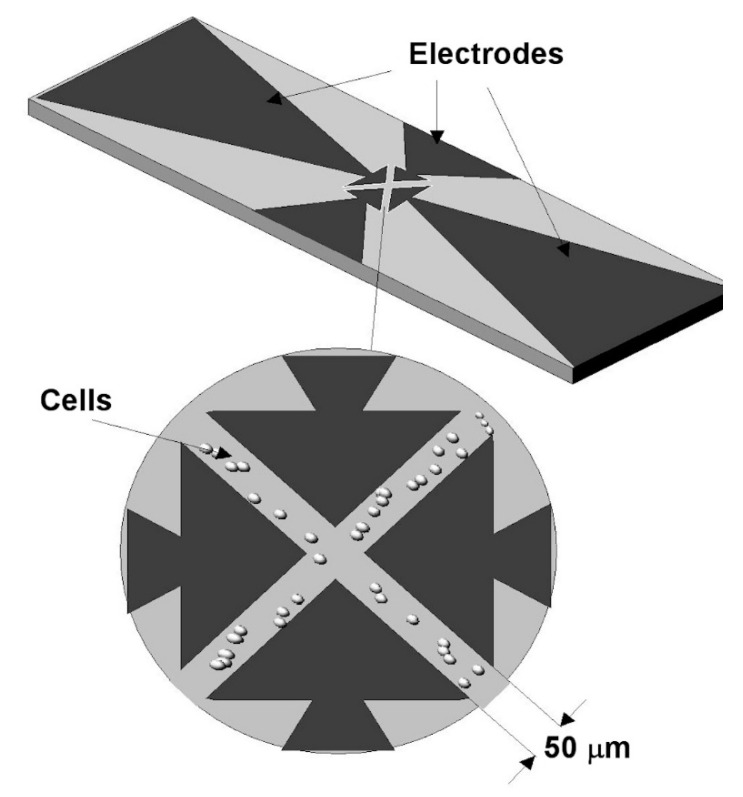
The makeup of the measurement chip containing an electrode quartet.

Electric and viscoelastic parameters of RBCs were measured by dielectrophoresis by means of an electric optical system of cell detection [22]. The procedures for optical registration are explained in details in the Appendix A, which includes the functional scheme of the electric optical system of cell detection (Figure A1) and the makeup of the measurement chip containing an electrode quartet (Figure A2). For the assay, 10 μL of whole blood from the vacutainer with citrate buffer was added to a 0.3 M sucrose solution (pH 7.36) in the 1:30 ratio.

The measurements were conducted immediately after the resuspension of RBCs. In a measurement cell, the RBCs were exposed to an NUAEF at the following settings: electric intensity, 105 V/m; electric intensity gradient, 10^11^ V/m^2^; and frequency range, 5 × 10^4^ to 1 × 10^6^ Hz. We analyzed cell polarizability, membrane capacity, electric conductivity, a position of crossover frequency, indices of aggregation and destruction of RBCs, velocity of cell motion toward electrodes, induced dipole moment, deformation amplitude of RBCs at a frequency of 10^6^ Hz, the deformation degree of the cells at a frequency of 5 × 10^5^ Hz, and summarized viscosity and summarized rigidity of RBCs. The data were processed using an original software suite, CELLFIND (developed by Federal Budgetary Research Institution “State Research Center of Virology and Biotechnology Vector”, Rospotrebnadzor; www.vector.nsc.ru). Intertest repeatability of the method was 7–12%.

### 2.3. Statistical Analysis

Statistical analysis of the data was performed in the SPSS software, v. 22 (www.ibm.com/spss). The Kolmogorov–Smirnov test was employed to characterize the distribution of numerical variables. In case of a normal distribution, the average (mean) and standard error of the mean (SEM) were calculated (presented as mean ± SEM). In the case of the normal distribution, the significance of differences (or correlations) of parameters was assessed by Student’s t test or Pearson’s correlation analysis. If a distribution was not normal, then nonparametric tests were performed (Mann–Whitney *U* test, Kolmogorov–Smirnov test, and χ^2^ test). In all the statistical analyses, the threshold of null hypothesis probability (*p*) was assumed to be 0.05. To evaluate the utility of RBC parameters for the stratification of severity of rheological disturbances in type 2 DM, receiver operating characteristic (ROC) analysis was carried out.

## 3. Results and Discussion

On the basis of the above-mentioned criteria, our patients with DM were distributed into two groups: a medium risk of damage to small vessels, i.e., medium severity of rheological and microcirculatory disturbances (Group 2; *n* = 47), and high risk of microcirculatory disturbances (Group 3; *n* = 15). The clinical parameters of the patients from these groups were found to be within the range corresponding to the risk of damage to large and small vessels.

The data on clinical examination and instrumental analyses of the groups are given in Table 1.

There were no significant differences in age, sex, alcohol consumption style, and cigarette smoking between the groups of patients with DM. By contrast, in the group with the high severity of rheological disturbances (Group 3), DM duration and arterial-hypertension duration were significantly greater and there was greater prevalence of (1) a family history of early cardiovascular diseases and (2) a history of ischemic heart disease with myocardial infarctions. Systolic and diastolic blood pressure and the prevalence of cardiac rhythm and conduction aberrations (*p* < 0.05) were higher in Group 3 than in Group 2. All patients with DM received sugar-lowering therapy (targeted to healthy levels of glucose metabolism control) and hypertension medication.

The patients with high severity of rheological disturbances showed worse manifestations of DM and its complications: this group featured higher levels of fasting glycemia (9.62 ± 0.69 versus 7.94 ± 0.33 mmol/L in Group 2, *p* = 0.018), glycated hemoglobin, albumin/creatinine ratio in spot urine, stronger manifestations of hyperlipidemia (mostly type 2B hyperlipidemia), and disturbances of purine metabolism, liver function, and the excretory function of kidneys.

It is worth mentioning that the patients with well-pronounced rheological disturbances (Group (3) had a higher body mass index, severer diabetic neuropathy and retinopathy (in 60% of these patients, we detected pre-retinopathy and proliferative retinopathy), angiopathy (in 53.3% of the cases, it corresponded to the IIB stage, when pain in lower extremities presented after walking less than 200 m, *p* < 0.0001).

The analysis of RBC parameters did not reveal significant differences among the groups in the RBC count, a color parameter, and hemoglobin. Nonetheless, it was found that patients with DM had higher hematocrit and greater RBC distribution width, whereas the average corpuscular volume and average hemoglobin content of RBCs turned out to be lower than those in the healthy controls (*p* = 0.047, *p* = 0.033, *p* = 0.002, and *p* = 0.009, respectively).

### 3.1. Assessment of Viscoelastic and Electric Parameters of RBCs in Patients with DM

**Figure 1:**
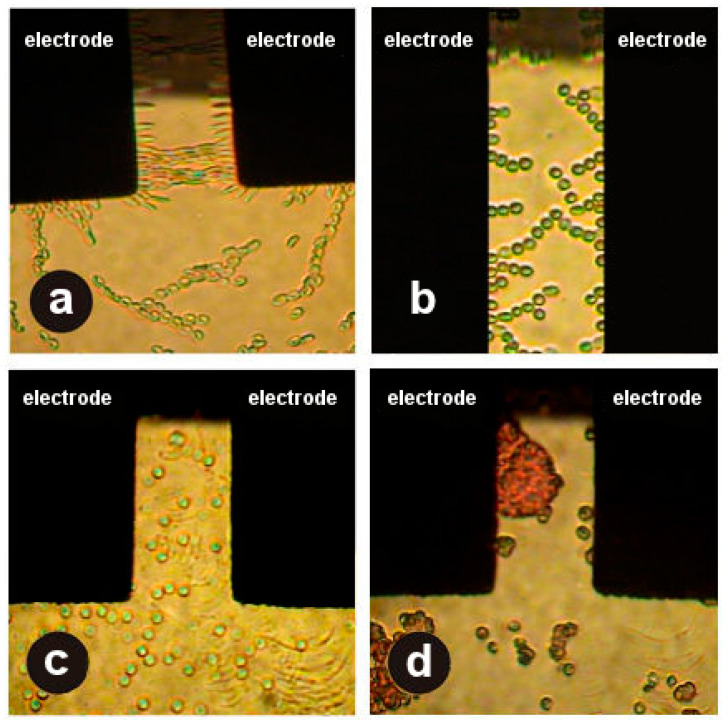
Behavior of red blood cells (RBCs) under the influence of the nonuniform alternating electric field (NUAEF) at a frequency of 10^6^ Hz (**a**) in the healthy control group and (**b**–**d**) in patients with diabetes mellitus (DM) (see the main text).

In the assay involving the NUAEF, RBCs of healthy controls (group 1) showed a high velocity of motion toward electrodes and high elasticity at frequencies of 5 × 10^5^ and 1 × 10^6^ Hz (Figure 1a). At low frequencies (5 × 10^4^ and 1 × 10^5^ Hz), we noted repulsion of the cells from the electrodes (negative dielectrophoresis) with hemolysis of some RBCs under the influence of the electric field.

In patients with DM, the behavior of RBCs in the NUAEF was substantially different: at high frequencies (5 × 10^5^ and 1 × 10^6^ Hz), deformability of the cells (m) and translational motion velocity of the cells (μm/s) toward the electrodes were found to be significantly lower (*p* < 0.01; Figure 1b). Pronounced destruction of RBCs was seen at all frequencies of the NUAEF (Figure 1c). Furthermore, the cells were prone to the formation of aggregates of various sizes (*p* < 0.02; Figure 1d).

There were differences in RBC characteristics associated with the severity of rheological disturbances in DM. In a solution of a dielectric, most RBCs from the patients with medium severity of rheological disturbances (Group 2) had a discocyte shape, and 24% ± 6% of the cells became spherocytes or assumed a shape of a “deflated ball.” In patients with severe microcirculatory disturbances (Group 3), we detected an increase in the proportion of nondiscocyte shapes up to 48% ± 6% (*p* < 0.001), with ~25% of the discocytes becoming spikelike. It is known that the membrane integrity and biconcave shape of RBCs are mostly ensured by the energy of high-energy compounds [23], primarily by various forms of ATP arising during glycolysis, which is the main pathway of energy metabolism in RBCs [24].

A decrease in the APT level in RBCs is accompanied by blockage of ion pumps, thereby leading to shifts in the ion balance in a cell–environment system. This process contributes to a reduction in the surface area/volume ratio of RBCs, to spherocytic transformation of RBCs, and to the emergence of spikelike protrusions. Such alterations impede the oxygen transport from the RBC to tissues and aggravate hypoxia, which stimulates fibrogenic phenomena with internal remodeling of vessel walls, thus driving the progression of diabetic angiopathy [13,24].

With the increasing severity of rheological disturbances (from Group 2 to Group 3 of patients with DM), there were increases in electric conductivity, indices of aggregation and destruction, summarized viscosity, and summarized rigidity. At the same time, deformation amplitude of RBCs, polarizability at 10^6^ Hz, dipole moment, electric membrane capacity of RBCs, and the velocity of cell motion toward the electrodes became significantly lower (*p* < 0.001–0.05; Table 2). The severity of microcirculatory disturbances in DM proved to be inversely related to deformation amplitude (*r* = −0.652, *p* < 0.0001) but directly correlated with summarized viscosity (*r* = 0.680, *p* < 0.0001) and summarized rigidity of RBCs (*r* = 0.635, *p* < 0.0001).

It is reported that the deformability of RBCs is determined by their viscoelastic characteristics [25]. According to our results, there was a decrease in RBC deformability with increasing summarized viscosity and summarized rigidity of these cells. These shifts may be associated with higher cholesterol content of the RBC membrane and a greater cholesterol/phospholipid index [26].

As a consequence of cholesterol exchange between RBCs and the lipoproteins adsorbed on their surface, cholesterol is incorporated into the RBC membrane. During this process, RBC size increases, and these cells change shape, with a substantial decrease in their filtration ability [27,28].

Such alterations cause microcirculatory disturbances and increase the risk of ischemic states including the diabetic foot. The decrease in cell elasticity under the influence of the NUAEF and progressive worsening of rheological disturbances in DM can be regarded as a “model” of RBC behavior at the level of capillaries, whose size is >2.5-fold less than the diameter of these cells [29]. On the one hand, “rigid and frail” RBCs, which are prone to aggregation, easily disintegrate under such conditions, whereas the aggregates damage the endothelial lining [27].

On the other hand, extensive regions of the capillary bed get “excluded” from the bloodstream and oxygen exchange because the aggregates and the rigid cells are unable to enter the zone of smallest vessels. The “rarefaction” of the capillary bed further aggravates hypoxia and ischemia at the periphery [30].

The propensity of RBCs for excessive disintegration and aggregation in patients with DM were found to correlate with low polarizability of the cells (*r* = −0.60, *p* = 0.02; *r* = −0.53, *p* = 0.04, respectively). Given that polarizability reflects biological activity of cells, its progressive decreases with increasing DM severity is possibly associated with activation of accelerated low-efficiency erythropoiesis in this disease. Activation of the renal juxtaglomerular apparatus, in conjunction with progressive narrowing of renal arteries, leads to increased production of erythropoietin, which is a stimulator of accelerated formation of RBCs [24]. Accelerated maturation of RBCs coincides with a peripheral release of the cells with altered membrane structure, including excessive presentation of carboxyl groups and a larger amount of structurally modified spectrin and phospholipid lysofractions. This state of affairs causes predominance (in the RBC population) of RBCs with lower resistance to various stressors. The changed structure of the RBC surface with signs of premature aging is a signal for immunocompetent cells to eliminate such RBCs from the bloodstream. During the destruction of RBCs, there is an intravascular release of intra-RBC ADP, ATP, and hemoglobin, which are potent inducers of the formation of aggregates, including mixed ones (leukocytic-thrombocytic-erythrocytic) [24,30]; this phenomenon can explain the high indices of destruction and aggregation at all frequencies of the NUAEF in our present study. Meanwhile, the increasing aggregation of RBCs is accompanied by the secretion (by the cell aggregates) of thromboplastic substances associated with the cell membrane, thereby creating a local hypercoagulative state. This process contributes to intravascular blood clotting and causes a secondary disorder of hemodynamics in microcirculation [11,31].

We revealed correlations of RBC characteristics with several clinical and biochemical parameters and some parameters of RBCs. It should be noted that most parameters of RBCs were found to correlate with the fasting glycemia level (*r* = −0.348, *p* = 0.005, for deformation amplitude at 10^6^ Hz; *r* = −0.266, *p* = 0.034, for the deformation degree at 5 × 10^5^ Hz; *r* = 0.439, *p* < 0.0001, for summarized viscosity; *r* = 0.338, *p* = 0.006, for summarized rigidity; and *r* = −0.32, *p* < 0.01, for average RBC diameter), with glycated hemoglobin (*r* = −0.551, *p* < 0.001, for deformation amplitude at 10^6^ Hz; *r* = −0.677, *p* < 0.0001, for the deformation degree at 5 × 10^5^ Hz; *r* = 0.601, *p* < 0.0001, for summarized viscosity; *r* = 0.63, *p* < 0.0001, for summarized rigidity; *r* = −0.49, *p* < 0.0001, for the average diameter of RBCs; *r* = −0.393, *p* < 0.001, for dipole moment; and *r* = 0.321, *p* < 0.01, for electric conductivity), the urine ratio of albumin to creatinine (*r* = −0.51, *p* < 0.0001, for deformation amplitude at 10^6^ Hz; *r* = −0.567, *p* < 0.0001, for the deformation degree at 5 × 10^5^ Hz; *r* = 0.517, *p* < 0.0001, for summarized viscosity; *r* = 0.451, *p* < 0.0001, for summarized rigidity; *r* = −0.482, *p* < 0.0001, for the average diameter of RBCs; *r* = −0.514, *p* < 0.0001, for dipole moment; and *r* = 0.247, *p* < 0.05, for electric conductivity). It is known that higher excretion of albumin with urine develops as a consequence of damage to (and dysfunction of) the renal vascular endothelium, increased pressure in the capillary network of glomeruli (glomerular hypertension), disruption of structural integrity of the glomerular basal membrane, and dysfunction of the canalicular epithelium [32,33].

Because the last two parameters reflect the compensation degree of carbohydrate metabolism, their relation with electric and viscoelastic characteristics of RBCs is not surprising. Glycated hemoglobin noticeably raises internal viscosity of RBCs (by forming cross-links with the cell membranes) and summarized rigidity too [34,35]. These effects appreciably influence the deformability of RBCs. It should be noted that the deformation amplitude of RBCs significantly correlated with DM duration and arterial-hypertension duration (*r* = −0.563, *p* < 0.01, and *r* = −0.42, *p* = 0.04, respectively).

The observed correlations indicate the involvement of RBCs in the pathogenesis of diabetic complications. The observed associations with lipid profile parameters (for high-density lipoprotein cholesterol: with deformation amplitude (*r* = 0.33, *p* = 0.013) and with summarized viscosity (*r* = −0.27, *p* = 0.043); for triglycerides: with deformation amplitude (*r* = 0.327, *p* = 0.008), with summarized viscosity (*r* = 0.325, *p* = 0.009), with summarized rigidity (*r* = 0.27, *p* = 0.03), and with cell polarizability at 10^6^ Hz (*r* = −0.875, *p* < 0.0001)) and with results of liver assays (for AST activity: with electric conductivity (*r* = 0.33, *p* = 0.008); for the total bilirubin level: with deformation amplitude (*r* = −0.283, *p* = 0.025), with summarized viscosity (*r* = 0.27, *p* = 0.032), and with summarized rigidity (*r* = 0.248, *p* < 0.05); for the albumin level: cell polarizability at 10^6^ Hz (*r* = 0.487, *p* = 0.028)) confirms interactions of RBCs with serum components. The most revealing are the correlations of RBC characteristics with the severity of diabetic retino- and angiopathy (stages of retino- and angiopathy respectively correlated with deformation amplitude at 10^6^ Hz (*r* = −0.31, *p* = 0.013, *r* = −0.377, *p* = 0.002), with the deformation degree at 5 × 10^5^ Hz (*r* = −0.228, *p* = 0.005, *r* = −0.414, *p* < 0.001), with summarized viscosity (*r* = 0.25, *p* = 0.046, *r* = 0.429, *p* < 0.0001), with summarized rigidity (*r* = 0.248, *p* = 0.048, *r* = 0.37, *p* = 0.003), with the average diameter of RBCs (*r* = −0.325, *p* = 0.009), and with dipole moment (*r* = −0.762, *p* < 0.0001, *r* = −0.454, *p* < 0.0001). Probably, the shifts in the structural and functional properties of RBCs contribute to the progression of DM complications, in agreement with data from other investigators [36,37].

We uncovered associations of the urine albumin/creatinine ratio with electric parameters of RBCs, namely, with dipole moment, which correlated with the value and density of the surface negative charge of RBCs. A dipole reflects spatial asymmetry of the distribution of electric charges throughout the cell volume. Traditionally, measurements of microalbuminuria and of the albumin/creatinine ratio in spot urine are used in the clinic for diabetic-nephropathy screening. It should be noted that in recent years, the microalbuminuria level gained popularity as a marker of plasma membrane functions of highly differentiated cells, including determination of the severity of endothelial dysfunction. Normally, due to the presence of a high negative charge on the endothelial-cell surface, negatively charged albumin does not cross the renal glomerular filter. To some extent, this charge is predetermined by the phospholipid structure in the cell membranes that include polyunsaturated fatty acids.

A decreased number of double bonds in the acyl residues of phospholipids corresponds to a lower negative charge, thereby causing excessive filtration of albumin into primary urine. These aberrations have been detected during the development of atherosclerosis; accordingly, microalbuminuria develops in hereditary hyperlipidemias, arterial hypertension, ischemic heart disease, or DM in patients with aberrant glucose tolerance.

In DM, there are changes of phospholipid structure in the membranes of highly differentiated cells (e.g., RBCs, endotheliocytes, and renal cells); this phenomenon influences the surface charge of the membranes. The reason for the tight link between electric parameters of RBCs and the albumin/creatinine ratio is obvious; this ratio is being evaluated as an indicator of the initial stages of endothelial dysfunction, which is one of the major components of DM pathogenesis [1,11].

It was found here that in patients with DM, there is a shift of crossover frequency to a high-frequency range (>5 × 10^5^ Hz; *p* < 0.01; Table 2). Such alterations are probably caused by the increased electric conductivity of RBCs in DM. The latter parameter reflects the ability of membranes to conduct an electric current and is substantially related to the altered membrane structure, in particular to high cholesterol content. It is known that an increase in the cholesterol content of membranes raises summarized viscosity and summarized rigidity of the RBC [26], limits lateral diffusion of receptors, and changes permeability to dissolved substances and ion transport, thus resulting in higher electric conductivity of the cell [13,24].

The progression of rheological-disturbance severity in our patients with DM coincided with a decrease in electric membrane capacity (*p* < 0.0001), and this effect may indirectly indicate membrane thickening. This phenomenon is probably related to several factors, for instance, the enhancement of mutual exchange of lipids with blood serum, as confirmed by correlations of RBC membrane capacity with the levels of total cholesterol (*r* = −0.51, *p* = 0.041) and triglycerides (*r* = −0.40, *p* = 0.03). Another possible contributor is adsorption of high-molecular-weight proteins on the surface of membranes (e.g., globulins, fibrinogen, and fibrin) in conjunction with the well-pronounced inflammation in patients with DM. This notion is supported by the higher levels of some inflammation markers observed in the present study (C-reactive protein, fibrinogen, the leukocyte count, and RBC sedimentation rate, as shown in Table 1).

With increasing severity of rheological disturbances, dipole moment was found to decrease in the patients with DM and to reach the lowest values in patients with high severity of microcirculatory disturbances (Group 3). This effect is a sign of a decreased electric charge of RBCs. On the one hand, a possible reason is the alteration of average RBC corpuscular volume in patients with DM (Table 1); this change—due to redistribution of membrane glycoproteins and glycolipids on the cell surface—lowered the density of the surface negative charge [23,24]. On the other hand, another possible explanation is a decrease in the absolute membrane concentrations of sialic and neuraminic acids, which account for ~60% of surface charge density [30]. Finally, the greater amounts of high-molecular-weight proteins (as a consequence of systemic inflammation in patients with DM) can “shield” the negative charge of RBCs, via adsorption on the surface of their membranes regardless of the antigen type. A decrease in the surface negative charge of RBCs increases their propensity for the formation of persistent aggregates [27,31].

Thus, it was demonstrated here that DM progression is accompanied by a process of RBC depolarization. Meanwhile, amplitude and frequency parameters of RBCs in the NUAEF were found to be accurate indicators of physicochemical properties of the membrane and cytoplasm of these cells as well as markers of their biological activity.

### 3.2. Cutoffs of RBC Parameters in DM Patients for Different Degrees of Rheological Disturbances

To assess the usefulness of RBC parameters for the stratification of severity of microcirculatory disturbances in patients with DM, ROC analysis was performed (construction of ROC curves) for the various characteristics of RBCs.

The most accurate discrimination between the two severity levels of rheological disturbances in DM was shown by summarized viscosity and summarized rigidity of RBCs, deformation amplitude of the cells at the frequency of 10^6^ Hz, the deformation degree of the cells at the frequency of 5 × 10^5^ Hz, dipole moment, and electric conductivity of the cells. Less accurate discrimination was manifested by the polarizability of RBCs at the frequency of 10^6^ Hz, morphology, structure of the RBC surface, and the velocity of RBC motion toward the electrodes (Table 3). Standard error for some parameters was in the range 0.018 to 0.093, and asymptomatic significance varied from 0.0001 to 0.168.

The results of combined evaluation of a “panel” of electric and viscoelastic parameters of RBCs in patients with type 2 DM for determining the severity of rheological disturbances are presented in Table 4, in comparison with the risk assessment criteria for vascular complications in patients with DM as proposed by the EASD. Our panel has relatively high sensitivity (97.8%), specificity (86.7%), positive predictive value (95.8%), negative predictive value (92.8%), and accuracy index (95.2%). The higher accuracy of evaluation of rheological disturbances when the totality of RBC parameters is used (as compared with individual assays of stand-alone electric and viscoelastic parameters of RBCs) suggests that a panel of viscoelastic and electric parameters of RBCs should be employed for this purpose in type 2 DM.

Our study has some limitations. Because of the experimental nature of this research, we recruited a relatively small number of subjects. Therefore, it is advisable to conduct an additional study on patients with type 2 DM featuring minimal rheological disturbances (recruitment of such patients is underway). Inclusion of such a group of patients with DM may increase the practical utility of this research because the rheological disturbances are reversible at the early stages.

A possible limitation of the proposed method of assaying RBC parameters is the necessity of taking into account various factors (alcohol, smoking, exposure to toxic compounds, an acute inflammatory process, and the acute stage of an infectious disease) that can affect these RBC characteristics.

The advantage of the proposed method over existing ones is that this diagnostic technique for rheological disturbances and diabetic microangiopathy allows to both identify this complication (regardless of the affected site) and determine its severity. The degree of changes in RBC parameters has the diagnostic value. Our method is barely invasive (involves only blood collection), thereby preventing serious complications in the patients and not requiring hospitalization. In relation to the physical and psychological state of a patient, there are no restrictions on our method. The evaluation of measured RBC parameters is objective and not affected by the qualifications and experience of the test operator. The dielectrophoresis assay requires only inexpensive widely available reagents thus increasing the accessibility of this method for a mass diagnostic program, including the one intended for screening. The proposed method is not labor-intensive and not costly. The high sensitivity, specificity, and clear-cut criteria for the evaluation of rheological-disturbance severity and diabetic microangiopathy are expected to facilitate diagnostics at an early stage of the disease.

## 4. Conclusions

Thus, our results on rheological disturbances in patients with type 2 DM lead to the following conclusions:

- worsening of rheological disturbances in patients with DM is accompanied by significant decreases in RBC deformation amplitude, dipole moment, polarizability at the frequency of 10^6^ Hz, and membrane capacity and corresponds to increases in electric conductivity, summarized viscosity, summarized rigidity, and propensity for hemolysis and aggregation;
- we identified the most useful parameters of RBCs for the stratification of severity of rheological disturbances in type 2 DM: summarized viscosity and summarized rigidity of RBCs, deformation amplitude of the cells at the frequency of 10^6^ Hz, the cell deformation degree at the frequency of 5 × 10^5^ Hz, dipole moment, and electric conductivity of the cells (area under the ROC curve: 0.713–0.982);
- the proposed panel of electric and viscoelastic parameters of RBCs has the following analytical characteristics: diagnostic sensitivity 97.8%; diagnostic specificity 86.7%; positive predictive value 95.8%, negative predictive value 92.8%, and accuracy index 95.2% when used as a diagnostic test, in comparison with the EASD risk assessment criteria for vascular complications in patients with DM.

The application of dielectrophoresis to the diagnosis and severity assessment of rheological disturbances and diabetic microangiopathy will enable rapid detection (with minimal expenses) of the DM complications that determine the prognosis, risk of disability, and life expectancy of the patients. This feature denotes unquestionable economic efficiency of the proposed diagnostic method.

## Figures and Tables

**Table 1:** Clinical and biochemical data in groups of patients with diabetes with varying degrees of risk of microcirculation disturbances (M ± m).

Clinical and Biochemical Indicators	Group 1, the Controls, *n* = 38	Group 2, with DM, Medium Risk of Microcirculatory Disturbances, *n* = 47	Group 3, with DM, High Risk of Microcirculatory Disturbances, *n* = 15
Age, years	48.4 ± 2.18	59.64 ± 1.18	57.8 ± 2.09
Gender (person)	F-24	F-35	F-8
	M-14	M-12	M-7
Body mass index (kg/m^2^)	27.52 ± 0.61	31.74 ± 0.82 **	35.62 ± 0.97 ***^
Diabetes experience (years)	-	8.2 ± 3.2	14.2 ± 2.7^
Duration of hypertension (years)	-	9.8 ± 4.3	15.9 ± 3.9^
Family history of early cardiovascular disease, *n*(%)	2 (5.3%)	18 (38.3%) ***	9 (60%) ***^^^
The stages of diabetic retinopathy, *n*(%)			
0—no	38 (100)	14 (29.8)	0
1—non-proliferative	0	24 (51.1)	6 (40)
2—preproliferative	0	8 (17.0)	6 (40)
3—proliferative	0	1 (2.1) ****	3 (20) ****^^^^
Stage diabetic angiopathy, *n*(%)			
0—no	38 (100)	0	0
1—I (asymptomatic)	0	24 (51.1)	0
2—II A	0	20 (42.6)	4 (26.7)
3—II B	0	3 (6.3)	
4—III	0	0 ****	
Glycosylated hemoglobin (HbAlc), %	4.78 ± 0.11	6.59 ± 0.025 ****	8.01 ± 0.07 ****^^^^
Albumin/Creatinine ratio in a single portion of urine, mg/mmoL	2.23 ± 0.06	3.34 ± 0.035 ****	4.1 ± 0.044 ****^^^^
Total Cholesterol, mg/dL	183.7 ± 5.9	208.48 ± 6.35 ***	217.5 ± 11.7 ***
HDL Cholesterol, mg/dL	51.43 ± 1.92	49.33 ± 1.52	42.46 ± 2.74 **^^
LDL cholesterol, mg/dL	77.38 ± 2.2	91.72 ± 2.8 **	112.46 ± 2.6 ***^^^
AST, U/L	12.52 ± 1.18	21.36 ± 1.26 *	28.53 ± 3.1 *^
Total bilirubin, µmol/L	11.88 ± 0.82	12.78 ± 1.4	19.24 ± 1.34 *^
Albumin, g/L	50.2 ± 1.2	49.1 ± 1.8	41.7 ± 0.94 *^
Triglycerides, mg/dL	126.08 ± 14.5	188.9 ± 27.7 *	222.3 ± 20.42 ***
Fasting blood glucose, mmol/L	5.76 ± 0.13	7.94 ± 0.33 ****	9.62 ± 0.69 ****^^
Uric acid (mg/dL)	360 ± 18	390 ± 15	420 ± 29
Creatinine, μmol/L	74.07 ± 1.58	74.94 ± 1.42	84.28 ± 5.43 **^^
Urea, mmol/L	4.5 ± 0.28	5.51 ± 0.31 **	6.04 ± 0.34 ***
C-reactive protein, mg/L	3.0 ± 0.8	3.6 ± 0.9	5.5 ± 0.7 **^^
Fibrinogen, g/L	2.4 ± 1.3	3.2 ± 1.8	5.1 ± 1.2 *^
The number of leukocytes, ×10^9^/L	4.56 ± 0.26	5.52 ± 0.42	6.75 ± 0.32
The number of red blood cells, ×10^12^/L	4.59 ± 0.096	4.63 ± 0.07	4.60 ± 0.17
Hemoglobin level, g/L	141.79 ± 2.25	140.93 ± 4.5	140.79 ± 2.25
Color indicator	0.92 ± 0.009	0.91 ± 0.008	0.87 ± 0.019 *
Hematocrit, %	42.41 ± 0.58	43.11 ± 0.64	44.79 ± 0.89 *
Mean corpuscular volume (MCV), fl	93.78 ± 0.52	92.89 ± 0.74	89.81 ± 1.46 ***^
Mean corpuscular hemoglobin (MCH), pg	31.21 ± 0.29	30.58 ± 0.31	29.4 ± 0.67 ***
Mean cell hemoglobin concentration (MCHC), g/L	333.11 ± 2.12	329.58 ± 2.2	326.4 ± 3.56
Red cell distribution width (RDW-CV), %	13.84 ± 0.08	14.11 ± 0.09 *	13.95 ± 0.18
Red cell distribution width (RDW-SD), fl	50.43 ± 0.36	50.17 ± 0.44	48.49 ± 0.65 ***^
RBC sedimentation rate, mm/h	10.20 ± 1.44	15.11 ± 1.58 *	19.8 ± 2.54 **^

Note: “M” is the mean, and “m” is the average error of the arithmetic average value;* significance (*p*) of differences from the control group (Group 1): * *p* < 0.05, ** *p* < 0.02, *** *p* < 0.01, **** *p* < 0.0001;^ significance (*p*) of differences from the Group 2: ^ *p* < 0.05, ^^ *p* < 0.02, ^^^ *p* < 0.01, ^^^^ *p* < 0.0001.

**Table 2:** Electrical and viscoelastic parameters of red blood cells in patients with diabetes with different degrees of microcirculatory disturbances (M ± m).

Electrical and Viscoelastic Parameters of Red Blood Cells	Group 1, the Controls, *n* = 38	Group 2, with DM, Medium Risk of Microcirculatory Disturbances, *n* = 47	Group 3, with DM, High Risk of Microcirculatory Disturbances, *n* = 15
The average diameter of RBCs, [μm]	7.55 ± 0.008	7.45 ± 0.011 ****	7.33 ± 0.013 ****^^^^
Deformation amplitude of RBCs at 10^6^ Hz, [m]	(8.16 ± 0.11) × 10^−6^	(6.18 ± 0.06) × 10^−6^ ****	(5.2 ± 0.04) × 10^−6^ ****^^^^
The Deformation degree of RBCs at 5 × 10^5^ Hz, (%)	73.1 ± 1.97	41.55 ± 1.15 ****	25.86 ± 1.34 ****^^^^
Summarized rigidity of RBCs, [N/m]	(5.57 ± 0.16) × 10^−6^	(8.62 ± 0.04) × 10^−6^ ****	(10.12 ± 0.03) × 10^−6^ ****^^^^
Summarized viscosity of RBCs, [Pa×s]	0.51 ± 0.009	0.70 ± 0.002 ****	0.78 ± 0.005 ****^^^^
Electric conductivity, [Cm/m]	(5.53 ± 0.11) × 10^−5^	(6.83 ± 0.21) × 10^−5^ ****	(7.96 ± 0.38) × 10^−5^ ****^^^
Electric membrane capacity, [F]	(7.3 ± 0.19) × 10^−14^	(4.44 ± 0.42) × 10^−14^ ****	(4.10 ± 0.74) × 10^−14^ ****
The velocity of RBC motion toward the electrodes, [μm/s]	7.74 ± 0.19	4.57 ± 0.18 ****	4.2 ± 0.34 ****
The position of crossover frequency, [Hz]	(5.5 ± 0.58) × 10^5^	(11.8 ± 2.2) × 10^5^ ****	(16.0 ± 6.0) × 10^5^ ***
Dipole moment, [Kl×m]	(12.4 ± 4.9) × 10^−22^	(7.22 ± 0.15) × 10^−22^ ****	(4.5 ± 0.25) × 10^−22^ ****^^^^
Polarizability of RBCs at 10^6^ Hz, [m^3^], ×10^−15^	0.574 ± 0.005	0.459 ± 0.017 ****	0.438 ± 0.005 ****
Index of RBCs destruction at 10^6^ Hz, (%)	0	2.4 ± 0.1	3.4 ± 0.53
Index of RBCs destruction at 5 × 10^5^ Hz, (%)	0	2.8 ± 0.4	3.11 ± 0.32
Index of RBCs destruction at 10^5^ Hz, (%)	0	2.3 ± 0.5	5.0 ± 0.4 ^
Index of RBCs destruction at 5 × 10^4^ Hz, (%)	0	2.0 ± 0.6	4.1 ± 0.32 ^
Index of RBCs aggregation, conventional units	0.62 ± 0.003	0.64 ± 0.005	0.65 ± 0.007 *

Notes: The magnitude of the dipole moment was determined at an electric field strength of 8.85 × 10^−12^ F/m. * significance (*p*) of differences from the control group (Group 1): * *p* < 0.05, ** *p* < 0.02, *** *p* < 0.01, **** *p* < 0.0001; ^ significance (*p*) of differences from Group 2: ^ *p* < 0.05, ^^ *p* < 0.02, ^^^ *p* < 0.01, ^^^^ *p* < 0.0001.

**Table 3:** Results of the analysis of ROC curves of erythrocyte indices in patients with diabetes by differentiating the degree of rheological disorders.

Electrical and Viscoelastic Parameters of Red Blood Cells	Area Under ROC Curve (AUC)	Asymptotic 95% Confidence Interval		Speci- Ficity, (%)	Sensi- Tivity, (%)
		Lower Bound	Upper Bound		
Summarized viscosity of RBCs	0.982	0.946	1.0	98.0	80
Deformation amplitude of RBCs at 10^6^ Hz	0.979	0.939	1.000	98.0	93.3
Summarized rigidity of RBCs	0.967	0.92	1.0	08.0	93.3
Dipole moment	0.950	0.884	1.000	98.0	93.3
The Deformation degree of RBCs at 5 × 10^5^ Hz	0.913	0.866	0.996	98.0	93.3
The average diameter of RBCs	0.890	0.98	0.982	77.6	86.7
Electric conductivity	0.713	0.565	0.861	83.7	86.7
Polarizability of RBCs at 10^6^ Hz	0.696	0.566	0.826	71.4	66.7
Erythrocyte surface structure	0.696	0.549	0.843	80.0	40.8
Altered RBC morphology	0.626	0.463	0.788	60.0	32.7
The velocity of RBC motion toward the electrodes	0.618	0.436	0.801	71.4	66.7

**Table 4:** Diagnostic performance of the panel of electric and viscoelastic parameters of RBCs in patients with type 2 DM for assessing rheological-disturbance severity, as compared with the risk assessment criteria for vascular complications in patients with DM according to the EASD.

Results from the Panel of Electric and Viscoelastic Parameters of RBCs	Results of Analysis Using Risk Assessment Criteria for Vascular Complications in DM (EASD)	
	Group of Medium Severity of Rheological Disturbances in DM, *n* = 47 Cases	Group of High Severity of Rheological Disturbances in DM, *n* = 15 Cases
Group of medium severity of rheological disturbances in DM, *n* = 48 cases	True positive: group of medium severity of rheological disturbances, *n* = 46	False positive: group of high severity of rheological disturbances, *n* = 2
Group of high severity of rheological disturbances in DM, *n* = 14 cases	False negative: group of medium severity of rheological disturbances, *n* = 1	True negative: group of high severity of rheological disturbances, *n* = 13

Calculation of the main characteristics of the method according to T. Greenhalgh [38].; Sensitivity 46: (46 + 1) × 100% = 97.8%; Specificity 13: (2 + 13) × 100% = 86.7%; Positive predictive value 46: (46 + 2) × 100% = 95.8%; Negative predictive value 13: (1 + 13) × 100% = 92.8%; Accuracy index (46 + 13)/(46 + 2 + 1 + 13) × 100% = 95.2%.

## Appendix A. The Procedure for Optical Registration

The procedure for optical registration

The electric optical system of cell detection allows to measure cell characteristics. It includes the following components: a computer, amplifier GZ-112/1, generator GSPF-052 coupled with the computer, a Micmed-6 microscope with a video camera, and a disposable measurement chip. A functional scheme of the electric optical system of cell detection is presented in Figure A1.

To monitor dielectrophoresis, a special measurement chip is used that is coupled with a light microscope. The measurement chip is illustrated in Figure A2. On one of its sides, chrome electrodes are created by photolithography. Between the electrodes, there is a small measurement chamber (10^−3^ × 54.10^−6^ × 0.2 × 10^−6^ m). Electrode thickness should be less than a cell radius (r_c_). This condition ensures exposure of the cell to a nonuniform electric field. The measurement chip is installed on a movable stage of a microscope.

The procedure for optical measurements consisted of the following major steps. The measurement chip was installed on the movable stage of the microscope and was fixed in place with clamps. The microscope objective was focused (Micmed-6, magnification 40–1600×) on the measurement chip.

Next, a cell suspension was injected between the electrodes of the measurement chamber and was covered with a coverslip. The microscope was focused on the cells (particles) located between the electrodes. After the cells came to a standstill, electrode voltage was switched on. Video capture software recorded a video file of kinetic movement of the particles between the electrodes via a digital Sony video camera (with a 4.5 mm charge couple device) installed on the microscope as an add-on photo compoment; the digital frame rate was 25 fps. The observation and data registration were carried out with back-lighting. The sequence of frames in the video file enables a researcher to measure the average particle size, to trace the path traveled by each particle separately, and to calculate its velocity. Measurement of the particle radius is done by means of a size standard (scale bar).

Snapshots and the video allow to determine the size, velocity of kinetic or rotary movement, deformation amplitude of a cell being examined, frequency regions of positive and negative dielectrophoresis, and a boundary between them: crossover frequency. On the basis of the experimentally determined parameters, the computer calculates summarized rigidity and viscosity of the cells, their electric capacity, dipole moment, induced charge, and polarizability of the cell. The experimentally determined parameters had been validated by metrological testing.

## References

[1] Fowler M.J. (2008). Microvascular and macrovascular complications of diabetes. Clin. Diabetes.

[2] Taylor R., Batey D. (2012). Handbook of Retinal Screening in Diabetes. Diagnosis and Management.

[3] Bowker J.H., Pfeifer M.A. (2008). Levin and O’Neal’s The Diabetic Foot.

[4] Krutikov E.S., Zhitova V.A., Tsvetkov V.A., Pol’skaja L.V., Polishchuk T.F., Krutikova M.S., Glushko A.S. (2015). Diagnostic Technique for Microangiopathy in Diabetic Patients. Patent RUS..

[5] Bakirci S., Celik E., Acikgoz S.B., Erturk Z., Tocoglu A.G., Imga N.N., Kaya M., Tamer A. (2019). The evaluation of nailfold videocapillaroscopy findings in patients with type 2 diabetes with and without diabetic retinopathy. North. Clin. Istanb..

[6] Uyar S., Balkarli A., Kazim Erol M., Yesil B., Tokuç A., Durmaz D., Görar S., Hilmi Çekin A. (2016). Assessment of the Relationship between Diabetic Retinopathyand Nailfold Capillaries in Type 2 Diabetics with a NoninvasiveMethod: Nailfold Videocapillaroscopy. J. Diabetes Res..

[7] Kaminska-Winciorek G., Deja G., Polańska J. (2012). Diabetic microangiopathy in capillaroscopic examination of juveniles with diabetes type 1. Postepy Hig. Med. Dosw..

[8] Maldonado G., Ríos C. (2017). Nailfold Capillaroscopy in Diabetes Mellitus: Potential Technique for the Microvasculature Evaluation. Endocrinol. Metab. Syndr..

[9] Chittenden S.J., Shami S.K. (1993). Microvascular investigations in diabetes mellitus. Postgrad. Med. J..

[10] Ma Y., Yang C., Tao Y., Zhou H., Wang Y. (2013). Recent technological developments in proteomics shed new light on translational research on diabetic microangiopathy. FEBS J..

[11] Cho Y.I., Mooney M.P., Cho D.J. (2008). Hemorheological disorders in diabetes mellitus. J. Diab. Sci. Technol..

[12] Schmid-Schonbein H., Lowe G.D.O. (1988). Fluid dynamics and hemorheology in vivo: The interaction of hemodynamic parameters and hemorheological “properties” in determining the flow behavior of blood in microvascular networks. Clinical Blood Rheology.

[13] Berk D.A., Hochmuth R.M., Waugh R.E., Agre P., Parker J.C. (1989). Viscoelastic properties and rheology. Red Blood Cell Membranes: Structure, Function, Clinical Implications.

[14] Dupire J., Socol M., Viallat A. (2012). Full dynamics of a red blood cell in shear flow. Proc. Natl. Acad. Sci. USA.

[15] Kruchinina M., Voevoda M., Peltek S., Kurilovich S., Gromov A., Kruchinin V., Rykhlitsky S., Volodin V., Generalov V. (2013). Application of optical methods in blood studies upon evaluation of severity rate of diffuse liver pathology. J. Anal. Sci. Meth. Ins..

[16] Kruchinina M.V., Gromov A.A., Generalov V.M., Kruchinin V.N., Shuvalov G.V., Minin O.V., Minin I.V. Dielectrophoresis erythrocytes images for predicting stroke recurrence based on analysis of hemorheological parameters. Proceedings of the International Symposium on Optics and Biophotonics VI; SPIE Saratov Fall Meeting 2018: Optical and Nano-Technologies for Biology and Medicine.

[17] Cefalu W.T. (2016). American Diabetes Association Standards of Medical Care in Diabetes-2016. J. Clin. Appl. Res. Ed..

[18] Rydén L., Grant P.J., Anker S.D., Berne C., Cosentino F., Danchin N., Deaton C., Escaned J., Hammes H.P., Huikuri H. (2013). The task force on diabetes, pre-diabetes, and cardiovascular diseases of the European Society of Cardiology (ESC) and developed incollaboration with the European Association for the study of diabetes (EASD). Eur. Heart J..

[19] Chew E.Y., Ambrosius W.T., Davis M.D. (2010). Effects of medical therapies on retinopathy progression in type 2 diabetes. N. Engl. J. Med..

[20] Baigent C., Landray M.J., Reith C., Emberson J., Wheeler D.C., Tomson C., Wanner C., Krane V., Cass A., Craig J. (2011). The effects of lowering LDL cholesterol with simvastatin plus ezetimibe in patients with chronic kidney disease (Study of Heart and Renal Protection): A randomised placebo-controlled trial. Lancet.

[21] Liewelyn H., Aun Ang H., Lewis K.E., Al-Abdullah A. (2014). Oxford Handbook of Clinical Diagnosis.

[22] Generalov K.V., Generalov V.M., Kruchinina M.V., Shuvalov G.V., Buryak G.A., Safatov A.S. (2017). Medical and biological measurements method for measuring the polarizability of cells in an inhomogeneous alternating electric field. Meas. Tech..

[23] Park Y., Best C.A., Popescu G., Johnson A.W., Harley. B.A. (2011). Optical Sensing of Red Blood Cell Dynamics. Mechanobiology of Cell-Cell and Cell-Matrix Interactions.

[24] Novitskiy V.V., Ryazantseva N.V., Stepovaya E.A. (2004). Fiziologiya i Patofiziologiya Eritrotsita [The Physiology and Pathophysiology of Red Blood Cells].

[25] Ju M., Leo H.L., Kim S. (2017). Numerical investigation on red blood cell dynamics in microflow: Effect of cell deformability. Clin. Hemorheol. Microcirc..

[26] Ercan M.D., Konukoglu S.O. (2002). The effects of cholesterol levels on hemorheological parameters in diabetic patients. Clin. Hem.-Orheol. Microcirc..

[27] Singh M., Shin S. (2009). Changes in erythrocyte aggregation and deformability in diabetes mellitus: A brief review. Indian J. Exp. Biol..

[28] Tomaiuolo G. (2014). Biomechanical properties of red blood cells in health and disease towards microfluidics. Biomicrofluidics.

[29] Vahidkhah K., Balogh P., Bagchi P. (2016). Flow of red blood cells in stenosed microvessels. Sci. Rep..

[30] Schiffman F.J. (2000). Pathophysiology of Blood.

[31] Li Q., Li L., Li Y. (2015). Enhanced RBC Aggregation in Type 2 Diabetes Patients. J. Clin. Lab. Anal..

[32] Wu H.Y., Huang J.W., Peng Y.S., Hung K.Y., Wu K.D., Lai M.S., Chien K.L. (2013). Microalbuminuria screening for detecting chronic kidney disease in the general population: A systematic review. Ren. Fail..

[33] Wu H.Y., Peng Y.S., Chiang C.K., Huang J.W., Hung K.Y., Wu K.D., Tu Y.K., Chien K.L. (2014). Diagnostic performance of random urine samples using albumin concentration vs ratio of albumin to creatinine for microalbuminuria screening in patients with diabetes mellitus: A systematic review and meta-analysis. JAMA Intern. Med..

[34] Kung C.-M., Tseng Z.-L., Wang H.-L. (2009). Erythrocyte fragility increases with level of glycosylated hemoglobin in type 2 diabetic patients. Clin. Hemorheol. Microcirc..

[35] Marini M.A., Fiorentino T.V., Andreozzi F., Mannino G.C., Succurro E., Sciacqua A., Perticone F., Sesti G. (2017). Hemorheological changes in adults with prediabetes detected by hemoglobin. A1c Nutr. Metab. Cardiovasc. Dis..

[36] Shin S., Ku Y. (2005). Hemorheology and clinical application: Association of impairment of red blood cell deformability with diabetic nephropathy. Korea-Aust. Rheol. J..

[37] Brown C.D., Ghali H.S., Zhao Z., Thomas L.L., Friedman E.A. (2005). Association of reduced red blood cell deformability and diabetic nephropathy. Kidney Int..

[38] Greenhalgh T. (2014). How to Read a Paper: The Basics of Evidence-Based Medicine.

